# Multinucleation of Incubated Cells and Their Morphological Differences Compared to Mononuclear Cells

**DOI:** 10.3390/mi10020156

**Published:** 2019-02-25

**Authors:** Shukei Sugita, Risa Munechika, Masanori Nakamura

**Affiliations:** Department of Engineering, Nagoya Institute of Technology, Nagoya 466-8555, Japan; mmm5ar07dd@gmail.com (R.M.); nakamura.masanori@nitech.ac.jp (M.N.)

**Keywords:** multinucleated cells, XTC-YF cells, morphological analysis, Y-27632, hydrophobic dish

## Abstract

Some cells cultured in vitro have multiple nuclei. Since cultured cells are used in various fields of science, including tissue engineering, the nature of the multinucleated cells must be determined. However, multinucleated cells are not frequently observed. In this study, a method to efficiently obtain multinucleated cells was established and their morphological properties were investigated. Initially, we established conditions to quickly and easily generate multinucleated cells by seeding a *Xenopus* tadpole epithelium tissue-derived cell line (XTC-YF) on less and more hydrophilic dishes, and incubating the cultures with medium supplemented with or without Y-27632—a ROCK inhibitor—to reduce cell contractility. Notably, 88% of the cells cultured on a less hydrophilic dish in medium supplemented with Y-27632 became multinucleate 48 h after seeding, whereas less than 5% of cells cultured under other conditions exhibited this morphology. Some cells showed an odd number (three and five) of cell nuclei 72 h after seeding. Multinucleated cells displayed a significantly smaller nuclear area, larger cell area, and smaller nuclear circularity. As changes in the morphology of the cells correlated with their functions, the proposed method would help researchers understand the functions of multinucleated cells.

## 1. Introduction

Most cell types normally have a single nucleus, whereas some types of cells contain multiple nuclei. Nuclear division without cytokinesis occurs in some types of mammalian cells, including megakaryocytes, which produce blood platelets, and some hepatocytes and heart muscle cells [1]. Cells of the monocyte/macrophage lineage can fuse and form large multinucleated giant cells that have long been recognized as a histopathological hallmark of tuberculosis, schistosomiasis and other granulomatous diseases [2]. Even mononuclear cells sometimes become multinucleated cells in culture [3]. 

Some studies indicate concerns of multinucleated cells to pathophysiological events. When endothelial cells obtained from sites of arteriosclerosis are cultured, they exhibit a multinucleated morphology [4]. Multinucleated cells are frequently seen in malignant neoplasms [5]. Multinucleated giant cells are found in epulis, during unusual patterns of chronic inflammation, and are responsible for eliminating foreign bodies and cell debris by phagocytosis [6]. 

The mechanism by which the properties of mononuclear cells are altered to a multinucleated morphology is not completely understood. Since researchers have attempted to use cells cultured in vitro for tissue engineering [7], understanding of the properties of multinucleated cells is important to produce risk-free tissues. 

The ultimate goal of the present study is to investigate the properties of multinucleated cells. For the purpose, we initially established a method to efficiently obtain multinucleated cells because people still struggle with harvesting multinucleated cells. The existence of multinucleated cells of NMFH-2 and NMFH-1 cells were 10.2% and 5.00%, respectively [5]. If experimental systems to collect many multinucleated cells are established, various biochemical, molecular and immunological assays can be made. Although a method designed to incubate multinucleated cells by exclusively selecting and sub-culturing these cells has been reported, the procedure took 10 months [3]. Zang et al. observed multinucleated cells after culture on a hydrophobic substrate in the absence of myosin [8]. In this study, we tested the administration of Y-27632, a ROCK inhibitor that decreases myosin activity and cell contractility, to efficiently obtain any type of cell without gene transfer. The cells were incubated on a less hydrophilic dish and cultured with Y-27632. After establishing the method for generating multinucleated cells, the nature of the multinucleated cells was evaluated based on the morphology of the cells and cell nuclei. This experiment is based on reports that alterations in the nuclear size and shape are associated with and diagnostic markers of human diseases, including cancer and other pathologies [9,10].

## 2. Materials and Methods 

### 2.1. Contact Angle of Dishes

Reportedly, multinuclear cells are more frequently observed after culture on a hydrophobic surface [8]. Thus, we initially assessed the hydrophobicity of a dish. The contact angle *θ* of the dish surface was measured to evaluate its hydrophobicity. A 5 μL water drop was placed on φ 35 mm plastic dish (430165, CORNING, Corning, NY, USA) and φ 35 mm glass bottom dish from Fine Plus International (FC27-10N, FPI, Kyoto, Japan) and Matsunami Glass Industry (D11140H, Matsunami, Kishiwada, Japan). After imaging the droplet from the lateral side of the dish with a digital camera (CX3, Ricoh Imaging, Tokyo, Japan), the radius *r* of the contact area and height *h* of the droplet was measured using image analysis software (ImageJ 1.48v, National Institutes of Health, Bethesda, MD, USA) as follows. First, ten points on the edge of the droplet were spotted manually and their coordinates (*x_i_*, *y_i_*) (*i* = 1, 2, 3, …, 10) were measured. Then, a circle that fits the measured points was calculated using the least squared method:

(1) $$\left(\begin{array}{c} A\\ B\\ C \end{array}\right)\;=\;{\left(\begin{array}{ccc} \sum x_{i}{}^{2} & \sum x_{i}y_{i} & \sum x_{i}\\ \sum x_{i}y_{i} & \sum x_{i}{}^{2} & \sum y_{i}\\ \sum x_{i} & \sum y_{i} & \sum 1 \end{array}\right)}^{-1}\left(\begin{array}{c} -\sum \left(x_{i}{}^{3}+x_{i}y_{i}{}^{2}\right)\\ -\sum \left(x_{i}{}^{2}y_{i}+y_{i}{}^{3}\right)\\ -\sum \left(x_{i}{}^{2}+y_{i}{}^{2}\right) \end{array}\right)$$

where *A*, *B*, and *C* are parameters of the circle. The equation of the circle is given by:

(2) $$x^{2}+y^{2}+Ax+By+C=0.$$

Finally, the radius *r* of the circle was determined as:

(3) $$r=\sqrt{\frac{A^{2}}{4}+\frac{B^{2}}{4}-C}.$$

The height of the droplet *h* was directly measured from lateral images of the droplet. The contact angle was calculated with the equation:

(4) $$\theta \;=\;2\arctan\frac{h}{r}$$

using the half angle method [11]. 

### 2.2. Cells

*Xenopus laevis* cells derived from tadpoles (XTC-YF, RCB0771, RIKEN BioResource Center, Tsukuba, Japan) were used for ease of handling. The cells were cultured at 25 °C in culture medium (Leibovitz’s L-15 Medium, Wako Pure Chemical Industries, Osaka, Japan) that had been diluted two-fold with sterilized distilled water. The medium included 10% fetal bovine serum (S1820, Biowest, Nuaillé, France) and a 1% antibiotic solution (P4333, Sigma-Aldrich, St. Louis, MO, USA).

### 2.3. Conditions Used to Prepare Multinucleated Cells

XTC-YF cells were seeded on the FPI and Matsunami glass bottom dishes to investigate the conditions required to generate multinucleated cells. Y-27632 (257-00511, Wako Pure Chemical Industries) was added to the culture medium at a concentration of 100 μM to suppress myosin-induced contraction.

The cultured cells were fixed with 10% neutral buffered formalin for 10 min followed by washes with phosphate-buffered saline (PBS(-)) to confirm the multinucleated phenotype. The cells were immersed in 32 μM Hoechst 33342 (Molecular Probes, Thermo Fisher Scientific, Tokyo, Japan) for 20 min to fluorescently stain the cell nuclei and washed with PBS(-). Phase contrast images of the cells and fluorescently stained cell nuclei were captured using an inverted fluorescence microscope (IX-71, Olympus, Tokyo, Japan) equipped with an EM-CCD camera (iXon Ultra 888, Andor Technology, Belfast, UK) through a 20× (UPLFLN20X, Olympus) or 40× (LUCPLFLN40X, Olympus) objective lens. 

For image analysis, 680 μm × 680 μm images at 20× magnification and 340 μm × 340 μm images at 40× magnification were captured. The image analysis software (ImageJ 1.48v) was used to create a superimposition of the phase contrast and fluorescence images. The total numbers of cells, *N_all_*, and multinucleated cells, *N_multi_*, in the images were counted. The percentage of multinucleated cells *R_multi_* was defined as follows:

(5) $$R_{multi}\;=\;\frac{N_{multi}}{N_{all}}\times 100\;[\%].$$

### 2.4. Time-Lapse Imaging

Time-lapse images were captured to directly observe cell division. The XTC-YF cells were seeded on the FPI glass bottom dish, as described in Section 2.3, and observed under an inverted microscope (IX-73, Olympus) equipped with a CCD camera (DP73, Olympus). The phase contrast images of the cells were captured through the 20× objective lens at 5 min intervals from 3 to 72 h after seeding. 

### 2.5. Morphometry of the Cells and Cell Nuclei

The XTC-YF cells were seeded on FPI glass bottom dishes, and half of the cultures were treated with 100 μM Y-27632. After a 48 h incubation, cells were fixed and stained as described in Section 2.3. The phase contrast images and images of the fluorescently labeled nuclei were captured using the setup described in Section 2.3. The images of the fluorescently labeled cell nuclei were binarized to obtain nuclear outlines. The cell shapes were manually outlined in the phase contrast image. From the outlines of the cells and nuclei, the cellular area *S_cell_* and nuclear area *S_nucleus_* were measured. In the case of multinucleated cells, the areas of individual nuclei were measured. The cellular (α*_cell_*) and nuclear (α*_nucleus_*) circularity values were measured using the following equations:

(6) $$\alpha_{cell}\;=\;\frac{S_{cell}}{M_{cell}{}^{2}}\times \frac{4}{\pi }$$

(7) $$\alpha_{nucleus}\;=\;\frac{S_{nucleus}}{M_{nucleus}{}^{2}}\times \frac{4}{\pi }$$

where *M_cell_* and *M_nucleus_* represent the major axis of the best fit ellipse of the cellular and nuclear areas, respectively. A circularity value of 1 represents a perfect circle and a value of 0 indicates a (segmented) line.

### 2.6. Statistical Analysis

The difference in contact angle between the dishes was determined using the Tukey method. The differences in morphological data between mononuclear and multinucleated cells were determined using unpaired *t*-tests. The data are presented as the means ± standard deviations (SD), and the significance level was set to *p* = 0.05.

## 3. Results and Discussion

### 3.1. Contact Angle of Glass Bottom Dishes

**Figure 1:**
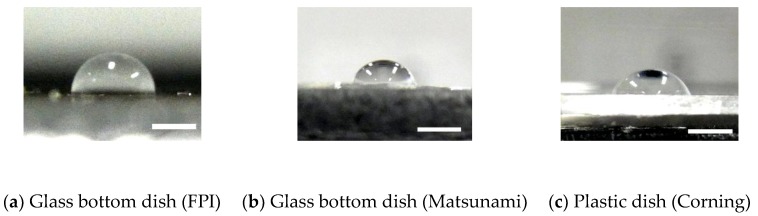
Typical images of droplets plated on (**a**) glass bottom dishes manufactured by Fine Plus International (FPI) and (**b**) Matsunami and (**c**) a plastic dish. Image contrast was enhanced for visibility. Bars correspond to 2 mm calibrated at the surface of the dish.

Images of droplets on dishes are shown in Figure 1. The glass bottom dish manufactured by FPI had a significantly larger contact angle (93 ± 2°, *n* = 10; Figure 1a) than the glass bottom dish from Matsunami Glass Industry (71 ± 4°, *n* = 10; Figure 1b) and plastic dishes (66 ± 3°, *n* = 10; Figure 1c). All groups had a significant difference in the contact angle. Based on these results, the dish manufactured by FPI is less hydrophilic than the dish manufactured by Matsunami Glass Industry and plastic dishes. Thus, in the following experiments, we used the glass bottom dish manufactured by FPI as a less hydrophilic dish and the dish from Matsunami Glass Industry as a more hydrophilic dish.

### 3.2. Comparison of Multinucleated Cells Plated on Different Dishes

**Figure 2:**
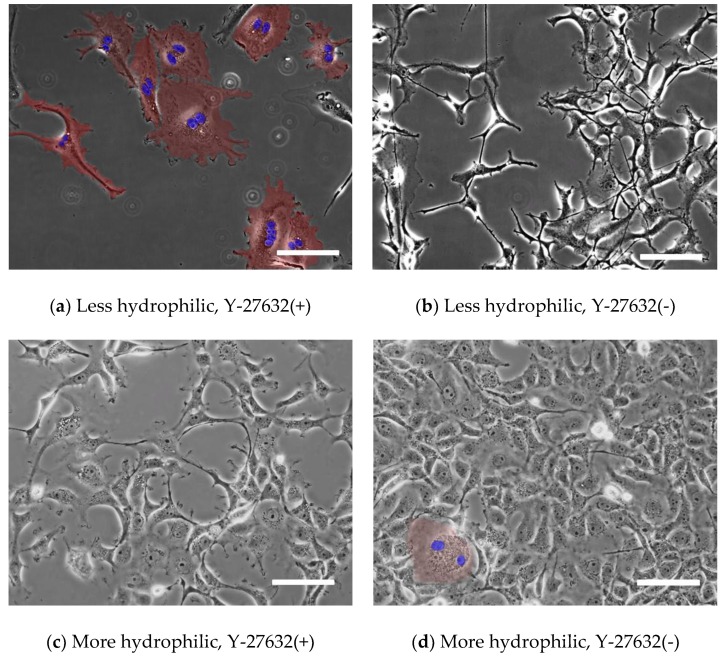
Typical images of XTC-YF cells seeded on (**a,b**) less hydrophilic (FPI) and (**c,d**) more hydrophilic (Matsunami) glass bottom dishes. Cells were incubated with (**a,c**) normal medium containing 100 µM Y-27632 and (**b,d**) normal medium alone. Multinucleate cells are shown in red and their nuclei are shown in blue. Scale bars represent 100 µm.

**Figure 3:**
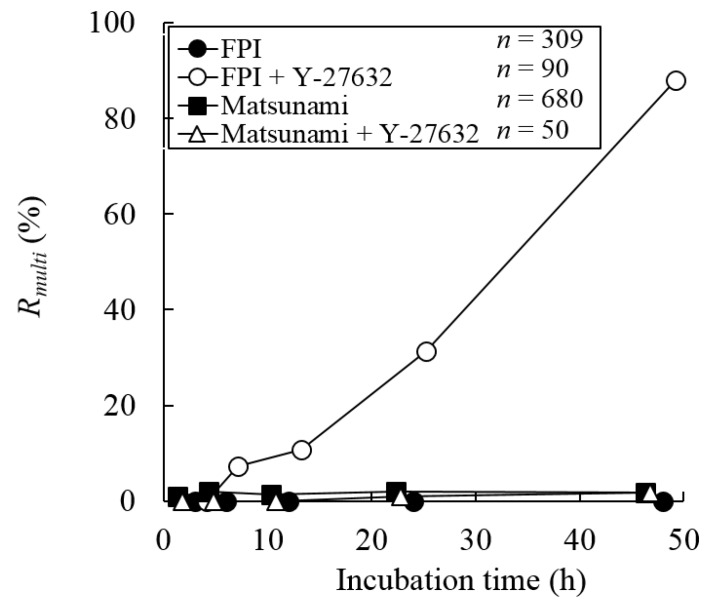
The percentage of multinucleated cells (*R_multi_*, as defined in Equation (5)) of XTC-YF cells after seeding on less (FPI) and more hydrophilic (Matsunami) dishes and culture in the presence or absence of Y-27632. *n*, Number of cells.

Figure 2 shows typical images of XTC-YF cells after the administration 100 µM Y-27632 at 48 h after seeding. Many multinucleated cells were observed after seeding on a less hydrophilic glass bottom dish (manufactured by FPI) and an incubation in medium containing Y-27632 (Figure 2a). On the other hand, when cells were seeded on a more hydrophilic dish (manufactured by Matsunami) or seeded on a less hydrophilic dish but incubated in normal medium, few multinucleated cells were observed (Figure 2b–d). Figure 3 plots the percentage of multinucleated XTC-YF cells (*R_multi_*) as a function of time after seeding. On the less hydrophilic glass bottom dishes, the *R_multi_* gradually increased, reaching 88% after 48 h. When the cells were seeded on the less hydrophilic dish without Y-27632 or on a more hydrophilic dish, the *R_multi_* was ≤ 5%, even after 48 h. Therefore, the condition required to produce multinucleated XTC-YF cells was incubation on a less hydrophilic dish in media containing 100 μM Y-27632. These results were in good agreement with a previous report showing that cells exhibited a multinucleated morphology after culture on a hydrophobic surface under conditions that inhibited myosin shrinkage [8]. Since Y-27632 is a Rho-kinase inhibitor that suppresses cellular contraction, the reagent is considered to inhibit cytokinesis. 

Multinucleated cells are produced at a high rate when the contractile force of myosin is inhibited and after culture on a relatively less hydrophilic dish. Unlike the long-term culture method [3], the present method yields multinucleated cells in the usual culture period. Additionally, unlike the gene transfer method [8], the present method is designed to study multinucleated cells in situ since the modification of gene expression is not required. The present method is thus beneficial for studying effects of multinucleation on cellular functions.

The surface hydrophobicity can be controlled by coating octadecyltriethoxysilane or fluorine resin-coating. The tuning of hydrophobicity for efficient production of multinuclear cells remain as future tasks. 

### 3.3. Changes in the Number of Nuclei during the Incubation

**Figure 4:**
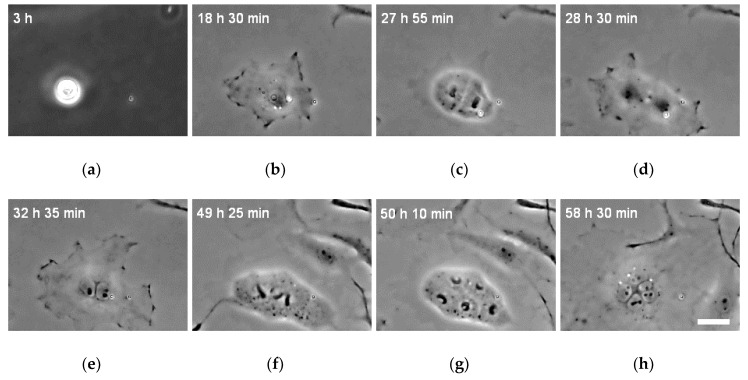
Typical time-lapse images of the XTC-YF cells incubated in medium containing 100 µM Y-27632 and seeded on a less hydrophilic glass bottom dish. Bars in (h) = 30 µm and are applicable to all images. The numbers in the upper left of the panels indicate the time after seeding.

Time-lapse images were captured to confirm that the multinucleated cells were not generated by fusion but instead through inefficient division. The time-lapse images of XTC-YF cells seeded on the less hydrophilic dish and cultured with medium containing 100 μM Y-27632 are shown in Figure 4 and Video S1. Over time, the projected area of the cells gradually increased (Figure 4b), and cells exhibiting a mononuclear morphology at the time of adhesion developed multiple nuclei following a subsequent cell division without cytokinesis (Figure 4c–e). Thus, the XTC-YF cells became multinucleated through inefficient division. The time of the first cell division after seeding was 21.5 ± 11.5 h. Some multinuclear cells divided again and the number of nuclei increased over time (Figure 4e–h). When multinucleated cells divided, all nuclei divided at the same time (Figure 4f). However, multinucleate cells with two nuclei sometimes divided into odd numbers of nuclei, such as three or five cell nuclei (Figure 4h). As shown in Figure 4 and Video 1, cells underwent multipolar mitosis and two clusters of chromosomes segregated into a single nucleus. This chromosomal mis-segregation is likely to produce aneuploid progeny [12], which is a condition associated with cancer [13,14].

**Figure 5:**
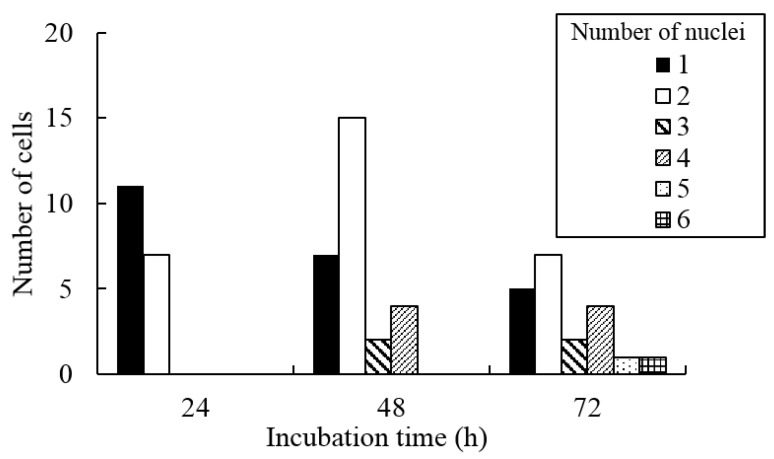
The number of nuclei in XTC-YF cells incubated with medium containing 100 µM Y-27632 and seeded on less hydrophilic glass bottom dishes.

Figure 5 shows the relationship between the elapsed time and the number of nuclei per cell. Forty-eight hours after seeding, many cells contained two nuclei. As shown in Figure 4 and Video 1, multinucleated cells further divided at later time points. This division increased the number of cells with more than two nuclei and decreased the number of cells with two nuclei at 72 h after seeding. 

### 3.4. Morphology of the Cells and Cell Nuclei of Multinucleated and Mononuclear Cells

**Figure 6:**
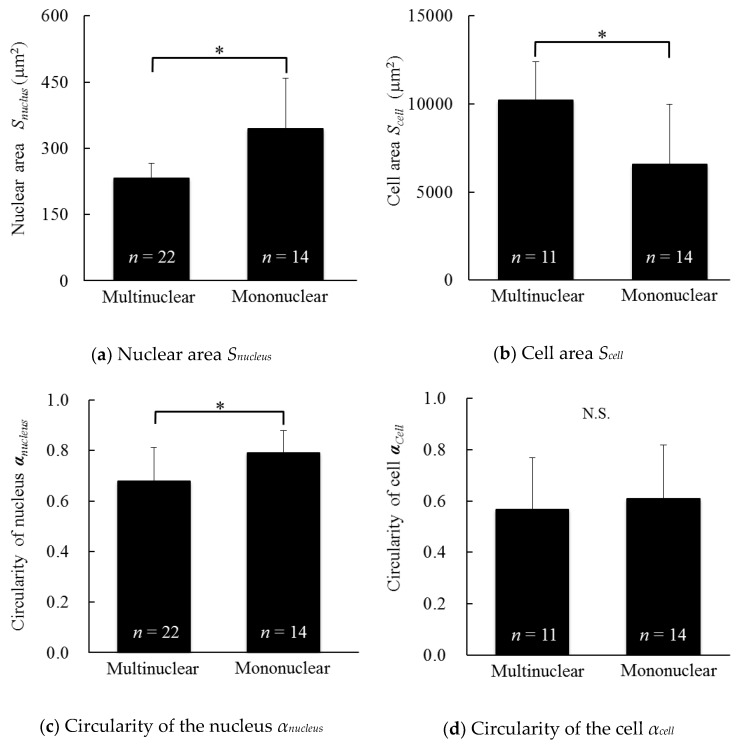
Morphology of multinucleated and mononuclear XTC-YF cells 48 h after seeding on the less hydrophilic glass bottom dish. Morphological data, such as (**a**) the nuclear area, (**b**) cell area, (**c**) circularity of the nucleus, and (**d**) circularity of the cell, are shown. *n*, Number of nuclei; *, *p* < 0.05.

Figure 6a shows the nuclear area (*S_nucleus_*) of multinucleated and mononuclear cells. The *S_nucleus_* of the multinucleated cells was significantly smaller than mononuclear cells. As confirmed in Video 1, chromosome division definitely occurred, and thus the smaller nuclei observed in multinuclear cells indicated chromosomal condensation. Since the nuclear size positively correlates with nuclear import rates and the concentrations of two transport factors, importin α and Ntf2 [15], the concentrations of these factors might be reduced in multicellular cells compared with mononuclear cells. Importin α and Ntf2 modulate the import of lamin B3 [15], a major component of the nuclear lamina that supports the nuclear envelope and is involved in DNA replication [16], suggesting a possible difference in DNA replication between multinucleated and mononuclear cells. 

A cell nucleus divided, as confirmed in the time-lapse movie (Video S1). Thus, normal numbers of chromosomes would be present in a single nucleus, even in the multinucleated cells. As shown in the results presented in Figure 6a, the *S_nucleus_* of the multinucleated cells was much smaller than mononuclear cells. Since the nuclear volume in cancer cells influences the proliferative activity [17], multinucleated cells might display differences in cell proliferation.

Figure 6b shows the cellular area (*S_cell_*) of multinucleated and mononuclear cells. The *S_cell_* of the multinucleated cells was significantly larger than mononuclear cells. In this study, Y-27632 was administered to obtain multinucleated cells. Since Y-27632 interferes with myosin II activity and reduces the tension of stress fibers, a cell treated with Y-27632 spreads and exhibits an increased cellular area [18]. Thus, the significant increase in the cell area of multinucleated cells might be due to the effect of Y-27632. Further experiments will be needed to determine whether Y-27632 or multinucleation increased the cell area.

According to Wilson, the ratio of the area of the cell nucleus to the area of the cell is a constant value [19]. If the ratio in multinucleated cells is defined for each nucleus within a cell, the ratio observed for multinucleated cells at 48 h after seeding (2.4 ± 0.6%) was smaller than mononuclear cells (5.9 ± 2.1%). However, multinuclear cells contain at least two nuclei. Thus, if the sum of the nuclear area in a single multinucleated cell is considered, the ratio is comparable to the nuclear area of mononuclear cells (5.0 ± 1.3%). In this sense, the ratio proposed by Wilson [19] is applicable to multinuclear cells. 

Figure 6c shows the nuclear circularity (α*_nucleus_*) of multinucleated and mononuclear cells. The nuclear circularity of the multinucleated cells was significantly smaller than mononuclear cells. The circularity of the nucleus tends to decrease in the presence of an abnormal number of chromosomes in cancer cells [20]. Thus, we speculated that the nuclei of multinucleated cells contain an abnormal number of chromosomes.

Figure 6d shows the cellular circularity (α*_cell_*) of the multinucleated and mononuclear cells. There were no significant differences in the cellular circularity between the multinucleated and mononuclear cells, and the cellular circularity appeared to be lower in the multinuclear cells. The cells treated with Y-27632 exhibited an increased degree of polarization and decreased circularity [21,22,23,24], or no change in cellular circularity [25]. Since Y-27632 was administered to obtain multinucleated cells in this study, the multinucleation of cells appears to decrease their circularity.

As shown in Figure 6, the morphology of the cell and cell nucleus of the multinucleated cells was completely different from the mononuclear cells. Since defects in nuclear size and shape are associated with and diagnostic markers of human disease, including cancer and other pathologies [9,10], further investigations focusing on cellular functions and their mechanisms will be required.

## 4. Conclusions

In summary, the present study established conditions to generate multinucleated XTC-YF cells, by seeding the cells on a less hydrophilic dish in a medium containing Y-27632. The present method quickly and easily produced multinucleated cells compared to pioneering methods [3,8]. Some cells divided to produce cells with an odd number of nuclei. The multinucleated cells had a significantly smaller nuclear area, larger cell area, and smaller nuclear circularity. The present method could contribute to improving our understanding of the nature of multinuclear cells. 

## Supplementary Materials

The following materials are available online at https://www.mdpi.com/2072-666X/10/2/156/s1, Video S1: Time-lapse images of cell division.
